# Hepatitis B, C, and D virus and human T-cell leukemia virus types 1 and 2 infections and correlates among men who have sex with men in Ouagadougou, Burkina Faso

**DOI:** 10.1186/s12985-018-1110-8

**Published:** 2018-12-29

**Authors:** Henri Gautier Ouedraogo, Seni Kouanda, Ashley Grosso, Rebecca Compaoré, Modibo Camara, Charlemagne Dabire, Rasmata Ouedraogo, Yves Traore, Stefan Baral, Nicolas Barro

**Affiliations:** 1 Biomedical and Public Health Department, Institut de Recherche en Sciences de la Santé (IRSS), Ouagadougou, 03BP7192 Burkina Faso; 2 University Ouaga1 Joseph Ki-Zerbo, Ouagadougou, Burkina Faso; 3 Institut Africain de Santé Publique, Ouagadougou, Burkina Faso; 4 Department of Epidemiology, Johns Hopkins Bloomberg School of Public Health, Baltimore, MD USA

**Keywords:** HBV, HCV, HDV, HTLV 1&2, MSM, Burkina Faso, West Africa

## Abstract

**Background:**

Men who have sex with men (MSM) are considered to be at significant risk for sexually transmitted infections (STI) and bloodborne viruses including viral hepatitis types B, C, and D (HBV, HCV, and HDV) and human T-cell leukemia virus types 1 and 2 (HTLV 1&2). This study aimed to assess the seroprevalence and correlates of HBV, HCV, HDV, and HTLV 1&2 antibodies among MSM in Ouagadougou, Burkina Faso.

**Methods:**

We conducted a cross-sectional survey to assess the biological and behavourial characteristics among MSM in Ouagadougou from January to April 2013. Serum specimens obtained were tested for the presence of HBV, HCV, HDV and HTLV-1&2 infections. MSM 18 years and older were recruited using respondent driven sampling (RDS). Population estimates and 95% confidence intervals (CI) adjusted for the RDS design were calculated using RDS Analysis Tool (RDSAT) version 6.0.1 (RDS, Inc., Ithaca, NY). Bivariate and multivariate logistic regression analyses were conducted to assess correlates of these infections using Stata 14.

**Results:**

A total of 329 MSM were tested. Prevalence was 20.4% (95% CI: 16.4–25.1) for HBV, 11.0% (95% CI: 8.0–14.8) for HCV, and 0.0% for HDV. Anti-HTLV 1&2 antibodies were found in 4.0% (95% CI: 2.3–6.8) of MSM. Factors independently associated with HBV infection were lack of condom use during the last anal sex act with a main male sexual partner and experience of condom tearing during anal sex. Presence of anti-HTLV 1&2 antibodies was associated with history of genital or anal lesions and injection drug use. None of the variables included in our study were associated with HCV.

**Conclusions:**

This study shows that HBV, HCV and HTLV 1&2 prevalence among MSM in Burkina is high and suggests that comprehensive STI prevention and sexual health education services for this group are needed.

## Background

Many men who have sex with men (MSM) are at a higher risk of contracting sexually transmitted infections (STI) and bloodborne pathogens [1–4]. These infections include Hepatitis B, C, and D viruses (HBV, HCV, and HDV) [5–12] and human T-cell leukemia virus types 1 and 2 (HTLV 1&2) [13, 14]. Hepatitis viruses are responsible for 96% of the 1.34 million deaths related to cirrhosis and liver cancer [15]. HBV and HDV transmission are due to the presence of the virus in biological fluids (blood, semen, and vaginal secretions) of the infected individual [16, 17].

HCV is mainly transmitted through blood via blood transfusion in developing countries and via sharing syringes for injection drug use in developed countries [18]. However, recent studies show there is sexual transmission of HCV among MSM [19]. Studies from Europe have shown an increase in HCV incidence among MSM. In Switzerland, for example, HCV annual incidence among MSM increased from 0.23 (95% Confidence Interval [CI]: 0.08–0.54) cases per 100 person-years in 1998 to 4.09 (95% CI: 2.57–6.18) cases per 100 person-years in 2011, compared to a change from 13.5 cases per 100 person-years to less than 1 case per 100 person-years among people who inject drugs [20].

HTLV is an infectious oncogenic retrovirus [13, 14] causing an aggressive cancer called adult T-cell leukemia/lymphoma (HTLV 1) [21] and neurological manifestations (HTLV 2) [13, 22]. HTLV 1&2 can be transmitted from mother to child, parenterally (through sharing needles or syringes), or sexually [23–25]. High morbidity of HTLV 1&2 has been found among populations at risk such as people who inject drugs and MSM [14, 26–28]. More than 20 million people are infected by HTLV 1&2 worldwide, and West Africa is considered a highly endemic area [23].

In Sub-Saharan Africa, very few data exist on hepatitis B, C, and D as well as on HTLV 1&2 epidemiology among MSM. This study aimed to assess the seroprevalence and correlates of hepatitis B, C and D virus and anti-HTLV 1&2 antibodies among MSM, a high-risk population in Burkina Faso.

## Methods

### Study design

A cross-sectional survey was conducted among MSM recruited using respondent driven sampling (RDS). RDS is a peer-recruitment sampling method designed to collect rigorous, representative data from hard-to-reach populations [29–31]. In preparation for this first integrated biological and behavioral survey, formative pre-survey research was conducted using formal meetings with MSM, local organizations, and government officials to explore MSM’s willingness to recruit their peers, challenges in finding diverse segments of this hidden population, and preferences expressed by MSM for all study procedures. The results of these meetings suggested that RDS should be used for the survey.

### Setting

The survey took place in Ouagadougou, the capital of Burkina Faso, a West African country with limited resources and high burden of viral hepatitis B and C.

### Study population and recruitment

MSM were eligible to participate if they meet the following criteria: (i) at least 18 years old, (ii) assigned male gender at birth, (iii) reported anal sex with a man at least once in the past 12 months, (iv) able to provide informed consent in French, Mòoré, or Dioula, (v) had a valid coupon, (vi) lived in Ouagadougou for at least the past three months, and (vii) agreed to complete a behavioral questionnaire and human immunodeficiency virus (HIV) testing.

Recruitment chains were initiated by MSM seeds. Six MSM seeds were purposely selected. Seeds were chosen who met the study eligibility criteria, represented diverse demographics (age, education, marital status, language, and HIV status), and who were willing to promote the study.

After giving informed consent, seeds were required to complete a questionnaire and have their blood drawn. These seeds were each provided with three coded coupons which were valid for four weeks to recruit MSM peers from their social network. Individuals who were recruited by seeds and enrolled in the study were then provided with three coded coupons for further recruitment of peers. This process continued until the target sample size was reached.

Participants received male condoms, condom-compatible lubricants, HIV education materials, and information regarding existing services. They also received 2000 XOF (~$4 United States dollars [USD]) for their time and transportation costs for each study visit. Per successfully eligible peer recruited to be part of the study (for up to three peers), recruiter had received 1500 XOF (~$3 USD). To avoid individuals participating multiple times, a single study office was used in addition to the use of a unique identification code. Study staff included a site manager, a coupon manager, two data collectors, an HIV and STI test counselor, and a lab technician. All staff were trained in the study procedures to avoid duplicate enrollments.

### Sample size

Sample size was previously estimated from an HIV behavior and seroprevalence study. The recruitment framework entailed 350 MSM. This size calculation was based on the assumption that populations who always use condoms have a 75% lower HIV prevalence than populations who do not, and the effectiveness of condoms is roughly 80%, with 73% used as a conservative estimate [32]. Overall, HIV prevalence was estimated at 15% with 19% prevalence among those who did not consistently use condoms. A design effect of 1.5 associated with RDS, significance level of 0.05, and a power of 80% was used.

### Data collection

Data were collected from January to April 2013 in Ouagadougou. Following informed consent, MSM participants completed interviewer-administered face-to-face questionnaires in a private room. Information included socio-demographic characteristics of participants, partnerships and sexual behaviors (with men and women), condom use, knowledge, attitudes and practices related to STI, and injection drug use.

### Samples collection and laboratory methods for testing

A trained nurse and lab technician respectively conducted HIV/STI counseling, venous blood specimen collection and HIV and syphilis testing in the study office. Serum samples were stored at − 20 °C et transported to the laboratory for hepatitis B, C, D and HTLV testing using DiaPro Enzyme Linked Immunoassay (ELISA) methods (https://www.diapro.it,). All tests were performed according to the manufacturer’s instructions.

### HBV testing

The ELISA test kit HBsAg One version ULTRA (ULTRA-Dia.Pro, Diagnostic BioProbes Srl, Italy), able to detect HBV “s” mutant was used for hepatitis B surface antigen detection. This test uses a mix of mouse monoclonal antibodies specific to determinants “a”, “d”, and “y” of HBsAg fixed to the surface of microwells. For test procedure, 150 μl of serum were added to the microwell together with 100 μl of a second mix of mouse monocolonal antibodies, conjugated with Horseradish Peroxidase (HRP) and directed against a different epitope of the determinant “a” and against “preS”. The specific immonocomplex, formed in the presence of HBsAg in the sample, is captured by the solid phase. At the end of of the one-step incubation for 120 min at 37 °C, microwells were washed to remove unbound serum proteins and HRP conjugate. Then 200 μl of chromogen/substrate (tetra-methyl-benzidine and hydrogen peroxidase, TMB/H2O2 mix) were added and incubate for 30 min at room temperature. In the presence of captured HBsAg immunocomplex, the colorless substrate is hydrolyzed by the bound HRP conjugate to a colored end-product. After blocking the enzymatic reaction by adding 100 μl of sulfuric acid, its optical density was measured with the ELISA reader (Reader 270, Microelisa system, Biomerieux, France) using 450 nm filter for raiding and a 620 nm filter for background subtraction. by the ELISA reader. The color intensity is proportional to the amount of HBsAg present in the sample.

### HDV testing

HBsAg reactive samples were subsequently tested for detection of anti-HDV antibodies using HDV Ab Dia.Pro, Diagnostic BioProbes Srl, Italy test kit, a competitive ELISA methods. The antibodies present in the serum compete with a virus-specific polyclonal IgG, labelled with peroxidase (HRP), for a fixed amount of rec-HDV coated on the microplate. The test was carried out with two steps incubation competitive system. First the 100 ul of sample were added to the plate and specific anti-HDV antibodies bind to the adsorbed antigen and incubated for 60 min at 37 °C. After washing (4–5 cycles), 100 μl of an enzyme conjugate antibody to HDV were added and binds to the free portion of antigen coated, following by second incubation for 60 min at 37 °C. After washing, 100 μl of chromogen/substrate mixture (TMB/H2O2 mix) was dispensed and incubated for 20 min at lab temperature (25°). The concentration of the bound enzyme on the solid phase becomes inversely proportional to the amount of anti-HDV antibodies in the sample and its activity was detected by the added chromogen/substrate. After blocking the enzymatic reaction by addition of 100 μl of sulfuric acid, the optical density was measured with the ELISA reader using a 450 nm filter for raiding and a 620 nm filter for background subtraction. The concentration of of HDV-specific antibodies in the sample is determined by means of a cut-off value that allowed for the semi-quantitative detection of anti-HDV antibodies.

### HCV testing

For anti-HCV antibodies, the fourth generation ELISA HCV Ab-Dia.Pro test (Diagnostics BioProbes Srl, Italy) was used. This test kit uses microplates coated with HCV-specific antigens derived from “core” and “n” regions encoding for conservative and immunodominant antigenic determinants (core peptide, recombinant NS3, NS4 and NS5 peptides). The solid phase was first treated with the diluted sample (200 μl diluent + 10 μl sample) and incubated à 37 °C, 45 min. HCV antibodies were captured, if present, by the antigens. After washing out all the other components of the sample, in the second incubation (45 min, 37 °C) bound HCV antibodies, immunoglobin G and M as well, were detected by addition of 100ul of polyclonal specific and hIgG&M antibodies, labelled with peroxidase (HRP). The enzyme captured with on the solid phase, acting on the chromogen/substrate mixture (100ul of TMB/H2O2), generates an optical signal that was proportional to the amount of anti HCV antibodies present in the sample. After incubation at lab temperature (25 °C) for 15 min, 100 μl of sulfuric acid were added into each well to stop the enzymatic reaction. The optical density was measured with the ELISA reader using 450 nm filter for raiding and a 620 nm filter for background subtraction. A cut-off value let densities was interpreted into HCV antibodies negative and positive results.

### HTLV 1&2 testing

Anti-HTLV 1&2 antibodies were tested using the ELISA HTLV 1&2 Ab-Dia.Pro ULTRA version (Diagnostic BioProbes Srl, Italy). This test uses microplates coated with HTLV1&2 specific synthetic immunodominant antigens derived from gp46–1, gp46-II and gp21-I. The solid phase was first treated with the sample (100 μl) and anti-HTLV 1&2 antibodies are captured during incubation (for 45 min at 37 °C) by the antigens coated on the microplate. After washing out all the other components of the sample, in the second incubation for 45 min at 37 °C, bound anti-HTLV 1&2 total antibodies was detected by the addition 100 μl of specific synthetic antigens derived from gp46–1, gp46-II and gp21, labelled with peroxidase (HRP). The enzyme captured on the solid phase, acting on the chromogen/substrate mixture, generates an optical signal that is proportional to the amount of anti HTLV 1&2 antibodies present in the sample. After blocking the enzymatic reaction, its optical density was measured by the ELISA reader (OD 450 nm).

For each testing, manufacturer negative and positive controls, and calibrator were used. The results were calculated by the means of a cut-off value (Co) determined on the mean optic density (OD) 450 nm value of the negative control as recommended in the kit manufacturer procedure. The test results were interpreted as positive when a ratio of the samples optic density at 450 nm (S) and the cut-of-value (Co) was superior to 1.1. Assay procedure was repeated for sample with equivocal result (S/Co = 0.9 to 1.1). All repeated testing provided negative or positive result and have been considered.

### Data processing

Data were entered using EpiData 3.1 (The EpiData Association, Odense, Denmark) and exported into Stata 14 (StataCorp, College Station, TX) for analysis.

Descriptive statistics were used to describe participants’ characteristics, HBV, HCV, HDV and HTLV 1&2 seroprevalence, and sexual behaviors. All proportions were adjusted to account for the RDS method. This adjustment takes into consideration the probability of each participant to be included in the study. This probability was measured through weighting based on the size of each participant’s network. Network size was determined using the survey question: “How many different people do you know personally who are MSM? i.e., you know them and they know you, you have seen them in the last 2 years, and you could contact them if you needed to?” The mean network size of study participants was 17 (minimum = 1 and maximum = 600). Population estimates and 95% CI adjusted for the RDS design were produced using the RDS Analysis Tool (RDSAT) version 6.0.1 (RDS, Inc., Ithaca, NY).

Bivariate and multivariate logistic regression analyses were conducted to assess factors associated with testing positive for each of the infections (HBV, HCV, HDV, and HTLV 1&2). Variables significantly associated with the infection at the *p* < 0.20 level were included in the multivariate models.

### Ethical considerations and protection of the participants

The study received ethical approval from the Ethics Committee for Health Research (CERS) of Burkina Faso. The study was implemented in a secure setting, and questionnaires were conducted in a private office. Collection of blood samples was performed by a trained staff member. Research ethic and sensitivity training were provided to all study staff before study implementation. Confidentiality was maintained by using a unique study identifier rather than real names on questionnaires and all study materials, protecting all electronic data with passwords, and storing hard copies of data in locked cabinets. Participant unique and anonymized codes were used to link study questionnaires with blood samples. All participants who tested positive for HIV were referred to a healthcare center.

## Results

A total of 329 sera of MSM including original seeds were tested for hepatitis B, C, and D virus and HTLV 1&2 infections.

### Socio-demographic characteristics

Participants’ socio-demographic characteristics are presented in Table 1. The mean age was 22.9 ± 4.0 years. In terms of sexual orientation, 71.9% (95% CI: 66.7–76.5) of MSM reported being gay/homosexual. Less than half of the study participants were bisexual (44.1%; 95% CI: 38.9–48.9). A minority (2.1, 95% CI: 0.0–4.4) were transgender. The majority of study participants were single (94.6, 95% CI: 91.6–96.5). Most of them were born in Burkina Faso. Over 70% (71.8, 95% CI: 66.8–76.4) of the participants recruited were students or pupils, while employees represented 21.6% (95% CI: 17.3–26.3).

**Table 1:** Characteristics of men who have sex with men study participants in Ouagadougou

Variables	n	RDS^a^-unadjusted %	RDS-adjusted % (95% CI^b^)
Current age (years)			
18–19	78	23.7	23.6 (19.3–28.6)
20–24	196	59.6	60.1 (54.7–65.3)
25–29	35	10.6	10.6 (7.7–14.5)
> =30	20	6.1	05.6 (3.6–08.7)
Total	329		
Country of birth			
Burkina Faso	272	82.7	82.7 (78.2–86.4)
Other countries	57	17.3	17.3 (13.6–21.8)
Total	329		
Childhood environment			
Urban	288	91.4	91.4 (87.8–94.1)
Rural	27	8.6	8.6 (5.9–12.2)
Total	315	100.0	
Highest educational level			
None or primary	25	7.6	7.6 (5.1–11.0)
Secondary	239	72.6	72.8 (67.7–77.4)
University	65	19.8	19.6 (15.7–24.3)
Total	329	100.0	
Occupation			
Student/pupil	236	71.7	72.0 (66.9–76.6)
Employed	72	21.9	2.15 (17.4–26.3)
Unemployed	21	6.4	6.5 (4.2–9.7)
Total	329		
Marital status (with a woman)			
Single	310	94.2	94.5 (91.5–96.5)
Other^c^	19	5.8	5.5 (3.5–8.5)
Total	329	100.0	
Number of biological children			
0	305	92.7	93.0 (89.7–95.3)
> =1	24	7.3	7.0 (4.7–10.3)
Total	329	100.0	
Sexual orientation			
Gay/homosexual	236	71.7	71.9 (66.7–76.5)
Bisexual	21	6.4	6.5 (4.42–9.7)
Heterosexual	72	21.9	21.6 (17.5–26.5
Total	329	100.0	
Anal sex position			
Receptive	19	5.8	5.7 (3.7–8.8)
Insertive	310	94.2	94.3 (91.2–96.3)
Total	329	100.0	
Number of male anal sex partners			
1	75	22.9	22.9 (18.6–27.8)
2	131	39.9	40.1 (34.9–45.5)
> =3	122	37.2	37.0 (31.9–42.4)
Total	328		
Past 12 month STI^d^ symptoms			
No	242	73.3	73.6 (68.6–78.1)
Yes	87	26.4	26.1 (21.6–31.1)
Don’t know	1	0.3	0.3 (0.0–2.2)
Total	329	100.0	
HIV^e^ status			
Negative	319	97.0	98.2 (96.7–99.0)
Positive	10	3.0	1.8 (1.0–3.3)
Total	329		
Ever injected drugs			
No	323	98.2	98.2 (96.0–99.2)
Yes	6	1.8	1.8 (0.8–4.0)
Total	329		

^a^*RDS* Respondent driven sampling

^b^*CI* Confidence interval

^c^Married or cohabiting, divorced, separated

^d^*STI* Sexually transmitted infection

^e^*HIV* Human immunodeficiency virus

### HBV, HCV, HDV and HTLV 1&2 seroprevalence

Tables 2 and 3 present the seroprevalence and factors associated with HBV, HCV and HTLV 1&2 in the bivariate and multivariate analyses.

**Table 2:** HBV^a^, HCV^b^ and HTLV^c^ 1&2 seroprevalence by demographic and behavioral characteristics among MSM^d^ and associated factors in Ouagadougou

Variables	n	HBV		HCV		HTLV 1&2	
		HBV positive	RDS^e^-adjusted HBV Positive (95% CI^f^)	HCV positive	RDS-adjusted HCV Positive (95% CI)	HTLV1&2 positive	RDS-adjusted HTLV1&2 Positive (95% CI)
Current age (years)							
18–19	78	17.9	17.7 (10.7–27.8)	7.7	7.3 (3.2–15.5)	3.8	3.9 (1.3–11.5)
20–24	196	19.9	19.6 (14.6–25.7)	13.3	13.3 (9.2–18.9)	4.1	4.1 (2.1–8.0)
25–29	35	22.9	23.1 (12.0–40.0)	2.9	02.9 (0.4–18.0)	0.0	–
> =30	20	35.0	35.9 (18.0–58.9)	15.6	16.4 (5.4–40.3)	10.0	10.9 (2.7–34.8)
Total	329	20.7	20.4 (16.4–25.1)	10.9	11.0 (8.0–14.8)	4.0	4.0 (2.3–6.8)
Country of birth							
Burkina Faso	272	21.0	20.7 (16.3–25.9)	9.9	09.9 (6.9–14.1)	4.0	4.1 (2.3–7.3)
Other countries	57	19.3	18.8 (10.7–31.1)	15.8	16.0 (8.5–28.1)	3.5	3.6 (0.9–13.2)
Childhood environment							
Urban	288	20.3	20.0 (15.8–25.0)	10.8	10.8 (7.6–14.9)	4.2	4.2 (2.4–7.3)
Rural	27	33.3	32.3 (17.5–51.7)	14.8	15.1 (5.7–34.0)	3.7	3.8 (0.5–22.6)
Highest educational level							
None or primary	25	24.0	24.4 (11.4–44.9)	4.0	4.1 (0.6–24.1)	4.0	4.1 (0.6–24.1)
Secondary	239	16.5	16.2 (12.0–21.4)	11.7	11.7 (8.1–16.4)	4.2	4.2 (2.3–7.7)
University	65	34.8	34.5 (23.9–46.7)	10.8	11.0 (5.3–21.4)	3.1	3.1 (0.8–11.8)
Occupation							
Student/pupil	236	18.1	17.9 (13.5–23.3)	12.3	12.2 (8.6–17.1)	3.8	3.8 (2.0–7.3)
Employed	72	28.8	28.4 (19.2–39.9)	5.6	5.7 (2.2–14.3)	2.8	2.9 (0.7–10.8)
Unemployed	21	22.7	21.2 (8.9–42.6)	14.3	14.3 (4.7–36.3)	9.5	9.5 (2.4–31.3)
Marital status (with a woman)							
Single	310	19.4	19.1 (15.1–23.9)	11.0	10.9 (7.9–15.0)	3.9	3.9 (2.2–6.8)
Other^g^	18	42.1	42.7 (22.8–65.3)	10.5	11.3 (2.8–35.7)	5.3	5.6 (0.8–31.2
Number of biological children							
0	305	20.8	20.5 (16.3–25.3)	10.5	10.5 (7.5–14.4)	3.9	4.0 (2.3–6.9)
> =1	24	20.8	20.1 (8.4–40.8)	16.7	17.6 (6.8–38.6)	4.2	4.4 (0.6–25.6)
Sexual orientation							
Gay/homosexual	236	20.3	20.1 (15.5–25.7)	10.2	10.3 (7.0–14.9)	3.0	3.0 (1.4–6.2)
Bisexual	21	31.8	30.5 (15.0–52.1)	19.0	19.0 (7.3–41.3)	4.8	4.8 (0.7–27.4)
Heterosexual	72	19.2	18.4 (11.1–29.0)	11.1	10.8 (5.4–20.3)	6.9	7.1 (3.0–16.0)
Anal sex position							
Receptive	19	26.3	26.9 (11.6–50.7)	10.5	10.8 (2.7–34.5	0.0	0.0
Insertive	310	20.5	20.1 (16.0–24.9)	11.0	11.0 (7.9–15.0)	5.0	5.0 (2.8–8.9)
Tested for STI^h^ in the last 12 months							
No	241	18.7	18.5 (14.1–24.0)	12.0	12.0 (8.4–16.8)	3.8	3.4 (1.7–6.6)
Yes	85	27.6	26.8 (18.5–37.1)	8.2	8.4 (4.0–16.6)	4.7	4.8 (1.8–12.2)
Past 12 month STI symptoms							
No	311	19.6	19.4 (15.4–24.3)	11.3	11.3 (8.2–15.3)	2.9	2.9 (1.5–5.6)
Yes	18	38.9	37.4 (18.6–61.0)	5.6	5.7 (0.8–31.4)	22.2	22.8 (8.8–47.4)
Vaccinated against HBV							
No	264	22.7	22.4 (17.8–27.9)	10.2	10.2 (7.1–14.5)	3.0	3.1 (1.5–6.0)
Yes	51	15.7	15.8 (8.1–28.6)	13.7	13.8 (6.7–26.4)	5.9	5.9 (1.9–16.9)
Don’t know	14	0.0	–	14.3	14.3 (3.6–42.9)	14.3	14.3 (3.6–42.9)
At least 2 sexual partners in the last 12 months							
Only males	268	17.9	17.7 (13.6–22.8)	10.8	10.9 (7.7–15.3)	4.5	4.5 (2.6–7.8)
Males and females	60	33.3	33.1 (22.3–46.0)	11.7	11.4 (5.4–22.2)	0.0	0
Type of sexual partners in the last 12 months							
Only males	201	19.9	19.8 (14.8–26.0)	12.9	12.9 (8.9–18.4)	2.0	2.0 (0.8–5.3)
Males and females	127	22.0	21.5 (15.2–29.5)	7.9	7.9 (4.3–14.1)	6.3	6.3 (3.2–12.2)
Ever experienced condom tearing during anal sex							
No	218	17.4	17.4 (12.9–23.0)	11.9	12.0 (8.3–17.1)	4.6	4.6 (2.5–8.4)
Yes	106	28.7	27.8 (20.1–37.0)	9.4	9.2 (5.0–16.4)	2.8	2.9 (0.9–8.6)
Condom use at last anal sex with a main male partner							
No	54	29.6	29.9 (19.1–43.4)	5.6	5.6 (1.8–16.1)	9.3	9.3 (3.9–20.6)
Yes	240	17.1	16.6 (12.4–21.9)	11.3	11.2 (7.8–15.9)	2.1	2.1 (0.9–5.0)
Condom use at last anal sex with a casual male partner							
No	34	17.6	17.9 (8.2–34.6)	11.8	11.9 (4.5–27.9)	8.8	8.9 (2.9–24.4)
Yes	224	19.6	19.2 (14.5–24.9)	10.3	10.4 (7.0–15.2)	3.6	3.6 (1.8–7.1)
HIV^i^ status							
Positive	10	40.0	40.0 (15.7–70.4)	10.0	11.0 (8.0–14.9)	0	0.0
Negative	319	20.1	20.1 (16.0–24.8)	11.0	10.0 (1.4–47.0)	4.1	4.1 (2.4–6.9)
Ever injected drugs							
No	323	20.7	20.5 (16.4–25.3)	11.0	11.2 (8.1–15.1)	3.7	3.8 (2.1–6.5)
Yes	6	16.7	16.7 (2.3–63.4)	0.0	–	16.7	16.7 (2.3–63.4)

^a^*HBV* Hepatitis B virus

^b^*HCV* Hepatitis C virus

^c^*HTLV* Human T-cell leukemia virus

^d^*MSM* Men who have sex with men

^e^*RDS* Respondent driven sampling

^f^*CI* Confidence interval

^g^Married or cohabiting, divorced, separated

^h^*STI* Sexually transmitted infection

^i^*HIV* Human immunodeficiency virus

**Table 3:** RDS^a^-adjusted prevalence and bivariate and multivariate logistic regression analyses of factors associated with HBV^b^ HCV^c^ and HTLV^d^ 1&2 infection among men who have sex with men in Ouagadougou

Variables	n	HBV infection					HCV infection					HTLV1&2 infection				
		Positive	OR^e^ (95% CI^f^)	*p*	AOR^g^ (95% CI)	*p*	Positive	OR (95% CI)	*p*	AOR (95% CI)	*p*	Positive	OR (95% CI)	*p*	AOR (95% CI)	*p*
Current age (years)																
18–20	78	17.7	1				07.3	1				3.9	1			
20–24	196	19.6	1.13 (0.57–2.23)	0.724	–		13.3	1.96 (0.76–5.04)	0.162	2.07 (0.80–5.33)	0.131	4.1	1.05 (0.27–4.10)	0.943	–	–
25–29	35	23.1	1.14 (0.52–3.75)	0.502	–		02.9	0.37 (0.04–3.33)	0.381	–	–	0.0	1			
> = 30	20	35.9	2.60 (0.86–7.87)	0.089	0.76 (0.11–4.94)	0.774	16.4	2.50 (0.55–11.20)	0.230	–	–	10.9	3.01 (0.46–19.65)	0.248	–	–
Total	329	20.4					11.0					4.0				
Country of birth																
Burkina Faso	272	20.7	1				09.9	1				4.1	1			
Other countries	57	18.8	0.88 (0.42–1.83)	0.742	–		16.0	1.73 (0.76–3.94)	0.186	1.88 (0.78–4.50)	0.153	3.6	0.86 (0.18–4.04)	0.853	–	–
Childhood environment																
Urban	288	20.0	1				10.8	1				4.2	1			
Rural	27	32.3	1.91 (0.80–4.51)	0.140	1.46 (0.53–3.98)	0.452	15.1	1.46 (0.47–4.55)	0.504	–	–	3.8	0.88 (0.11–7.17)	0.910	–	–
Highest educational level																
None or primary	25	24.4	1				04.1	1				4.1	1			
Secondary	239	16.2	0.60 (0.22–1.62)	0.319	–		11.7	3.11 (0.40–24.17)	0.277	–	–	4.2	1.04 (0.13–8.58)	0.970	–	–
University	65	34.5	1.58 (0.54–4.57)	0.393	–		11.0	2.90 (0.33–25.23)	0.332	–	–	3.1	0.76 (0.06–8.93)	0.830	–	–
Occupation																
Student/pupil	236	17.9	1				12.2	1				3.8	1			
Employed	72	28.4	1.84 (01.0–3.40)	0.051	1.64 (0.60–4.47)	0.331	05.7	0.43 (0.14–1.29)	0.134	0.22 (0.03–1.38)	0.109	2.9	0.73 (0.15–3.51)	0.700	–	–
Unemployed	21	21.2	1.07 (0.36–3.36)	0.907			14.3	1.19 (0.32–4.34)	0.784			9.5	(0.52–13.16)	0.238	–	–
Marital status																
Single	310	19.1	1				10.9	1				3.9	1			
Other*	18	42.0	3.03 (1.16–7.86)	0.023	1.44 (0.30–6.80)	0.640	11.3	1.03 (0.22–4.72)	0.964	–	–	5.6	1.79 (0.21–14.88)	0.589	–	–
Number of biological children																
0	305	20.5	1				10.5	1				4.0				
> = 1	24	20.1	0.98 (0.98–2.78)	0.972	–		17.6	1.82 (0.58–5.73)	0.299	–	–	4.4	1.11 (0.14–9.04)	0.921	–	–
Sexual orientation																
Gay/homosexual	236	20.1	1				10.3	1				3.0	1			
Bisexual	21	30.5	1.58 (0.57–4.31)	0.370	–		19.0	2.05 (0.63–6.64)	0.229	–	–	4.8	1.62 (0.18–13.96)	0.661	3.31 (0.22–47.9)	0.378
Transgender	72	18.4	0.90 (0.46–1.78)	0.782	–		10.8	1.05 (0.44–2.48)	0.904	–	–	7.1	2.47 (0.75–8.11)	0.133	2.29 (0.27–19.0)	0.440
Anal sex position																
Receptive	19	26.9	1				10.8	1				0.0				
Insertive	310	20.1	0.67 (0.23–1.97)	0.477	–		11.0	1.02 (0.22–4.65)	0.979	–	–	5.0	–	–	–	–
Past 12 month STI symptoms																
No	311	19.4	I				11.3	1				2.9	1			
Yes	18	37.4	2.47 (0.91–6.73)	0.075	1.03 (0.24–4.32)	0.964	05.7	0.47 (0.06–3.72)	0.478	–	–	22.8	9.76 (2.65–35.89)	0.001	14.19 (2.88–69.8)	0.001
Vaccinated against HBV																
No	264	22.4	1				10.2	1				3.1	1			
Yes	51	15.8	0.65 (0.28–1.46)	0.299	–		13.8	1.41 (0.57–3.46)	0.450	–	–	5.9	5.25 (0.50–7.81)	0.324	–	–
Don’t know	14	1	–				14.3	1.46 (0.30–6.95)	0.630	–	–	14.3	5.25 (1.00–27.70)	0.051	2.02 (0.28–14.34)	0.477
Type of sexual partners in the last 12 months																
Only males	201	19.8	1				12.9	1				2.0	1			
Males and females	127	21.5	2.29 (1.22–4.30)	0.010	220 (0.83–5.78)	0.108	07.9	0.57 (0.26–1.25)	0.164	0.98 (0.39–2.40)	0.977	6.3	3.27 (0.96–11.19)	0.058	3.13 (0.92–10.64)	0.068
Ever experienced condom tearing during anal sex																
No	218	17.4	1				12.0	1				4.6	1			
Yes	106	27.8	1.81 (1.04–3.16)	0.035	2.37 (1.21–4.67)	0.012	09.2	0.74 (0.34–1.61)	0.454	–	–	2.9	0.61 (0.16–2.29)	0.467	–	
Condom use at last anal sex with a regular male partner																
No	54	29.9	1				05.6	1				9.3	1			
Yes	240	16.6	0.46 (0.23–0.92)	0.029	0.41 (0.18–0.93)	0.033	11.2	2.13 (0.61–7.36)	0.230	–	–	2.1	0.21 (0.06–0.75)	0.017	0.24 (0.03–1.77)	0.163
Condom use at last anal sex with a casual male partner																
No	34	17.9	1				11.8	1				8.9	1			
Yes	224	19.2	1.09 (0.42–2.81)	0.857	–		10.3	0.86 (0.27–2.68)	0.795	–	–	3.6	0.38 (0.05–1.53)	0.175	0.50 (0.08–3.10)	0.456
HIV status																
Positive	10	40.0	1				11.0	1				0.0	–			
Negative	319	20.1	0.37 (0.10–1.38)	0.141	0.35 (0.06–1.85)	0.218	10.0	1.10 (0.13–9.11)	0.923	–	–	4.1	–	–	–	–
Ever injected drugs																
No	323	20.5	1				11.2					3.8	1	–	–	–
Yes	6	16.7	0.78 (0.08–6.83)	0.819	–		0.0	–	–	–		16.7	5.11 (0.55–47.77)	0.152	7.19 (1.12–46.25)	0.038

^a^*RDS* Respondent driven sampling

^b^*HBV* Hepatitis B virus

^c^*HCV* Hepatitis C virus

^d^*HTLV* Human T-cell leukemia virus

^e^*OR* Odds ratio

^f^*CI* Confidence interval

^g^*AOR* Adjusted odds ratio

### Factors associated with HBV

The prevalence of HBV among MSM was estimated at 20.4% (95% CI: 16.4–25.1). Bivariate analyses showed that the factors associated with HBV infection were reporting multiple sexual partnerships with at least two male partners and a female sexual partner (*p* = 0.010), experiencing condom tearing during anal sex (*p* = 0.035), not using a condom at last anal sex with a main male sexual partner (*p* = 0.029), and being divorced, widowed, married or cohabiting with a woman (*p* = 0.023). Multivariate analysis showed that only not using a condom at last anal sex with a main male sexual partner (*p* = 0.033) and experiencing condom tearing during anal sex (*p* = 0.012) were independently associated with HBV infection (Table 3).

### Seroprevalence of HCV

The prevalence of HCV among MSM is estimated at 11.0% (95% CI: 8.0–14.8). Although patterns of disparity were observed according to some characteristics of MSM, bivariate and multivariate analyses with study variables did not reveal any factors associated with HCV infection among MSM (Table 3).

### Seroprevalence of HDV

Anti-HDV antibody testing was performed on HBV-positive samples. No participants tested positive for anti-HDV antibodies, meaning that the seroprevalence was 0.0%.

### Seroprevalence and correlates of anti-HTLV 1&2 antibodies

The results show that the seroprevalence of the HTLV virus in the MSM surveyed is estimated at 4.0% (95% CI: 2.3–6.8). In the bivariate analysis, the factors associated with HTLV 1&2 infection were a history of STI symptoms (genital lesions) during the last 12 months (*p* = 0.001), not using a condom at last anal sex with a main male sexual partner (*p* = 0.017), and not using a condom at last anal sex with a casual male sexual partner (*p* = 0.018). Only the history of genital lesions (penis or anus) during the last 12 months and injection drug use were independently associated with the seroprevalence of anti-HTLV 1&2 antibodies in the multivariate analysis (Table 3).

## Discussion

To our knowledge, this study is the first in West Africa to report the seroprevalence of hepatitis B, C, and D and anti-HTLV 1&2 antibodies among MSM. It shows that the seroprevalence of HBV and HCV were very high and that of anti-HTLV 1&2 antibodies was relatively high.

### HBV

The prevalence of HBV (20.4%) in our sample of MSM is higher than those reported among other populations in Burkina Faso. In a recent systematic review in Burkina Faso covering the period from 1996 to 2017, the authors estimated the prevalence of HBV at 9.41% among the general population, 11.11% among pregnant women, 11.73% among blood donors and 12.6% among people living with HIV [33]. Our results suggest that MSM are a vulnerable group for HBV infection. Modes of transmission other than vertical transmission may explain the high prevalence of HBV among MSM [7, 11, 12]. In this study, the factors associated with HBV infection in the bivariate analysis were for the most part, factors related to sexual behavior, including multiple sexual partnerships with male and female partners, in addition to age ≥ 30 years and marital status with a woman. Older age had already been identified in some studies as associated with HBV infection [6, 7, 34], and could be explained by the accumulation of risk of exposure over time and vaccination against HBV coverage, which is increasing among younger individuals. Vaccination against HBV was only introduced in Burkina Faso in 2006 for infants [33].

The majority of MSM in our study identified themselves as gay, but over 44% reported being bisexual and have sex with men and women, and some are in a conjugal relationship with women. Bisexual MSM could have a higher risk of transmitting and acquiring STI [35], which could explain the higher prevalence of HBV infection among married MSM.

Multivariate analysis in our study showed that only condom use at last anal sex with a main male sexual partner (*p* = 0.033) and condom tearing during anal sex (*p* = 0.012) were independently associated with HBV infection, suggesting transmission of the virus through high-risk sex.

### HDV

In our study, no HBsAg-bearing MSM was infected with HDV. A prior study found a 0.1% (95% CI: 0.06–0.23) HDV prevalence among men in general population of Burkina Faso [36]. The very low prevalence in our study could be explained by the small size of the sample. However, although the number of positive HBsAg samples analyzed in our study is low, zero seroprevalence of anti-HDV antibodies has also previously been reported by studies in Sub-Saharan Africa. An analysis of 49 pregnant women with HBsAg in Burkina Faso revealed no evidence of anti-HDV antibodies [37]. Similarly, in Mozambique, Cunha et al., in their study of 146 blood donors infected with HBV, found no positive cases of anti-HDV antibodies [38].

### HCV

West Africa is considered an area with a high prevalence of HCV [39, 40]. We found in our study a prevalence of 11% in MSM. This prevalence is three times higher than that reported in Burkina Faso, and in West Africa in general. An analysis of the blood samples collected during the 2010 Demographic and Health Survey in the Burkinabe population estimated the HCV prevalence at 3.6% [41]. Systematic reviews have also estimated the pooled prevalence of HCV in the West African population between 3 and 5% [39, 42]. HCV transmission is mainly through blood, especially through transfusion with contaminated blood or nosocomial transmission with contaminated material [18]. Among MSM, there is often an increased risk of infection from injection drug use and HIV infection [8, 9, 43, 44]. In a recent systematic review, the authors found that the prevalence of anti-HCV antibodies was estimated at 40 and 6.7%, respectively, among HIV-positive MSM who were injection drug users and non-drug users [45]. Furthermore, traumatic anal sex may be involved in HCV transmission among MSM.

### HTLV 1&2 seroprevalence

HTLV 1&2 viruses are probably among the most neglected bloodborne viruses among MSM in Africa, and data are very scarce. This first study of MSM in Burkina Faso shows that the overall seroprevalence of anti-HTLV 1&2 antibodies was 4%. This seroprevalence is higher than those reported among pregnant women in West Africa, especially in Burkina Faso in 2006 (1.02%), in Côte d’Ivoire in 1989 (1.9%), and in Ghana in 2006. (2.1%) [23]. More recently in Nigeria, Yuguda et al. reported a seroprevalence of 3.2% among blood donors in Nigeria, with all positive cases being male [46]. The high prevalence of anti-HTLV 1&2 antibodies in MSM in our study may be explained by risk behaviors [26, 47]. The factors independently associated with the presence of anti-HTLV 1&2 antibodies in our study were a history of genital lesions (penis or anus) during the last 12 months and injection drug use. Other authors have found that HTLV infection in MSM is associated with the presence of STI including syphilis or genital lesions [14, 48, 49], or injection drug use. In a study in Argentina among people who inject drugs, MSM, sex workers, and tuberculosis patients, the authors found an overall prevalence of anti-HTLV 1&2 antibodies of 2.4%, and the highest prevalence (19.1%) was among people who inject drugs [27].

Our study has a number of limitations. Participants may have underreported sensitive behaviors, including injection drug use, which is punishable by law in Burkina Faso. The study was cross-sectional, therefore it is unknown whether the identified risk factors preceded the associated infections. Also, in this seroprevalence study, the testing was based on serological methods using fourth generation ELISA. Even these methods have very high specificity and sensitivity, a positive HBsAg or HCV and HTLV antibody test does not confirm the stage of infection during the study. Finally, there are specific limitations to the RDS method. The RDS financial incentive, while modest, may encourage more low-income MSM to participate in the study and lead to selection bias. Despite these limitations, RDS was identified during the preparatory phase of data collection as the best approach to recruiting MSM in this context. In addition, our study provides the first seroprevalence data for hepatitis B, C, and D and HTLV viruses among MSM in Burkina Faso. These data fill a gap and will help map the epidemiology of these infections among high-risk populations in Africa.

## Conclusions

This first study shows that MSM in Burkina Faso are heavily affected by hepatitis B and C viruses and HTLV infection. However, no MSM in this study tested positive for HDV. Sexual behavioral factors, genital or anal lesions, and injection drug use may play an important role in the spread of these viruses among MSM, particularly HBV and HTLV. These results highlight the need to strengthen the prevention of STI generally among MSM, but specifically the prevention of HBV transmission through vaccination. Sexual health education, strengthening accessibility of condoms and condom-compatible lubricants, and integrated STI services in HIV programs for MSM should be considered. Similarly, interventions to increase awareness of the risks associated with injecting drugs should be undertaken among MSM to limit the spread of HTLV.

## Abbreviations

AOR: Adjusted odds ratio
CERS: Ethics Committee for Health Research of Burkina Faso
CI: Confidence interval
ELISA: Enzyme Linked Immunoassay
HBsAg: Hepatitis B virus surface Antigen
HBV: Hepatitis B virus
HCV: Hepatitis C virus
HDV: Hepatitis D virus
HIV: Human immunodeficiency virus
HRP: Horseradish peroxidase
HTLV 1&2: Human T-cell leukemia virus types 1 and 2
Ig: Immunoglobilin
MSM: Men who have sex with men
OR: Odds ratio
RDS: Respondent driven sampling
RDSAT: RDS Analysis Tool
TMB/H2O2: Tetra-methyl-benzidine/hydrogen peroxyde
USD: United States dollars
